# Supplementary material for: Detection of the thietane precursor in the UVA formation of the DNA 6-4 photoadduct
Source: Nat Commun. 2020 Jul 17;11:3599. doi: 10.1038/s41467-020-17333-y (PMC7368040; doi:10.1038/s41467-020-17333-y)
Supplement: Supplementary file 1 — Supplementary Information [file 41467_2020_17333_MOESM1_ESM.pdf]

Supplementary Information

Detection of the thietane precursor in the UVA formation of the DNA 6-4 photoadduct

Ortiz-Rodríguez et al.

Supplementary Figures and Tables

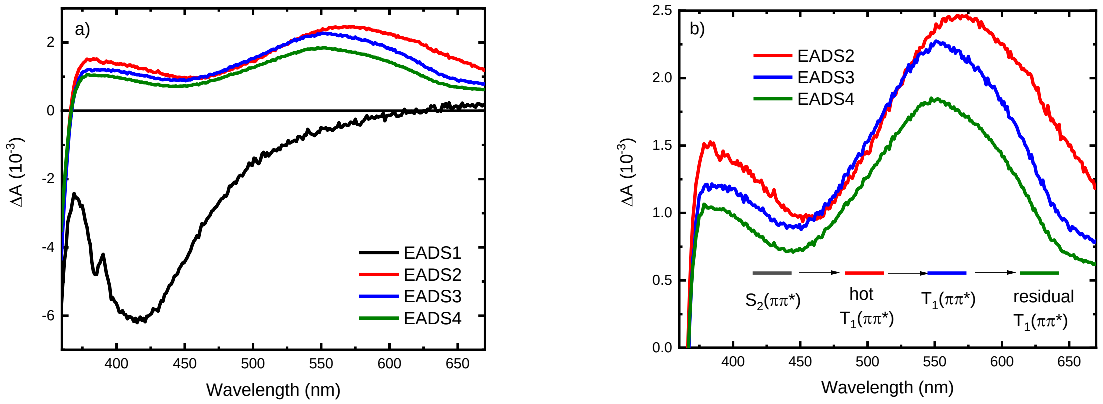

Supplementary Figure 1. (a) Evolution associated difference spectra (EADS) for 4tT extracted from the global and target analysis of the fs-transient absorption data. (b) Zoom-in of the last three EADS. Residual stimulated Raman signal from the solvent is observed at ca. 389 nm.

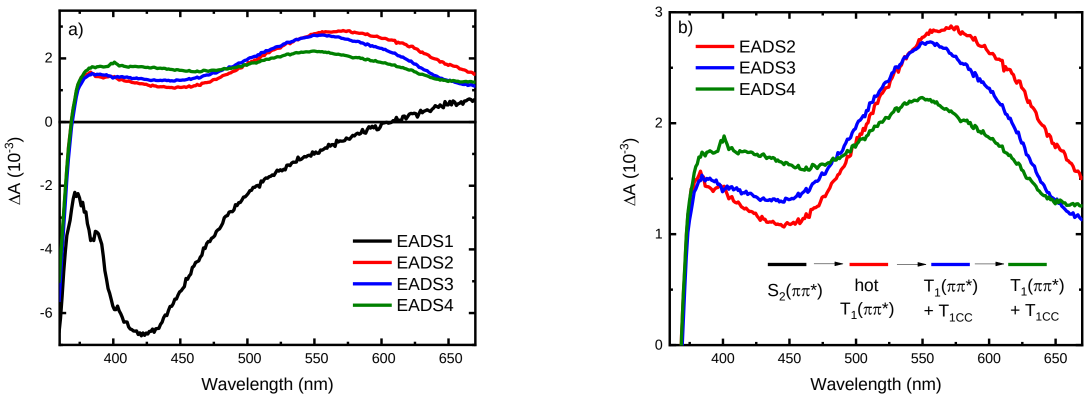

Supplementary Figure 2. (a) Evolution associated difference spectra (EADS) for $T(T4tT)_2T_2$ extracted from the global and target analysis of the fs-transient absorption data. (b) Zoom-in of the last three EADS. Residual stimulated Raman signal from the solvent is observed at ca. 389 nm. Note that the T_{1CC} spectrum is contributing to the amplitude around 430 nm during both EADS3 and EADS4, as observed in Figure 4e.

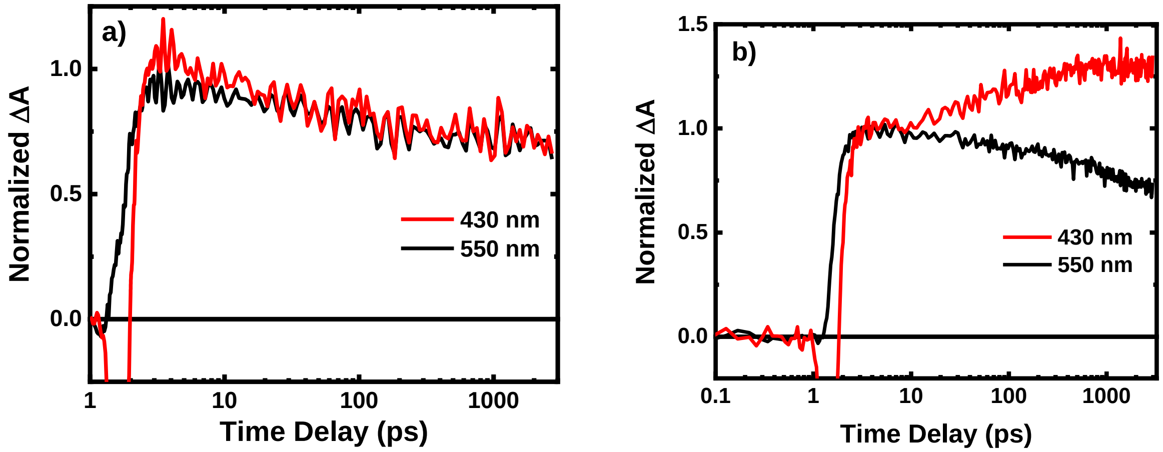

Supplementary Figure 3. Normalized decay traces of (a) 4tT and (b) $T(T4tT)_2T_2$ at 430 and 550 nm probe wavelengths.

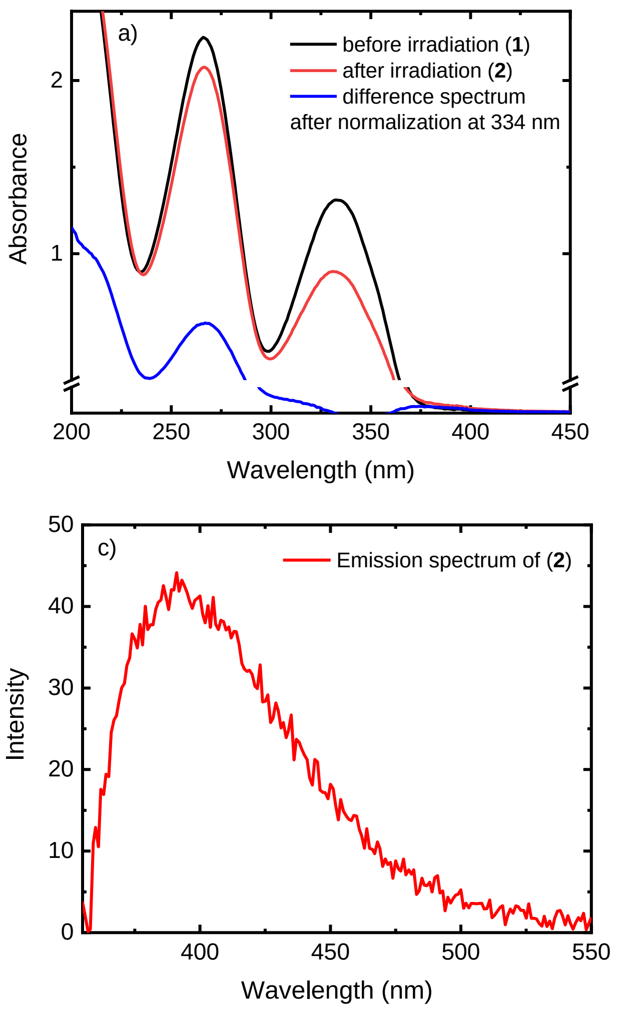

Supplementary Figure 4. (a) Absorption spectra of T(T4tT)$_2$T$_2$ before and after laser irradiation at 342 nm. The oligonucleotide was irradiated with 0.75 mW of power for a period of 35 minutes in PBS pH 7.4. This corresponds to an irradiation dose of 202 J cm^{-2}. The difference absorption spectrum is the subtraction of the absorption spectrum after laser irradiation and the absorption spectrum before irradiation, both normalized at 334 nm. In the difference spectrum, note the absorption with maximum intensity around 320 nm that extends to ca. 400 nm, as observed in the actual absorption spectrum of the (6-4) photoadduct (see Figure 4a in ref. 2).[1] The intensity below zero in the difference spectrum is due to a slightly over-subtraction for the 4tT chromophore using the normalization method. (b) Excitation spectra of the (6-4) photoadduct in PBS pH 7.4. The excitation spectrum was collected at the emission wavelength of 390 nm. The (6-4) photoadduct contains a 5-methyl-2-pyrimidone moiety (Pyr) that is associated with the absorption band around 320 nm. The emissive properties of the (6-4) photoadduct comes from the Pyr chromophore.[2-7] The emission observed in T(T4tT)$_2$T$_2$ is consistent with those previously reported for Pyr and the (6-4) photoadduct (Supplementary Figure 4c).[1] Thus, in addition to the excitation spectra collected after irradiation, this result further shows that the (6-4) photoadduct was formed. (c) Emission spectrum obtained after irradiation of T(T4tT)$_2$T$_2$ at 342 nm. The excitation wavelength used for the emission spectrum was 320 nm.

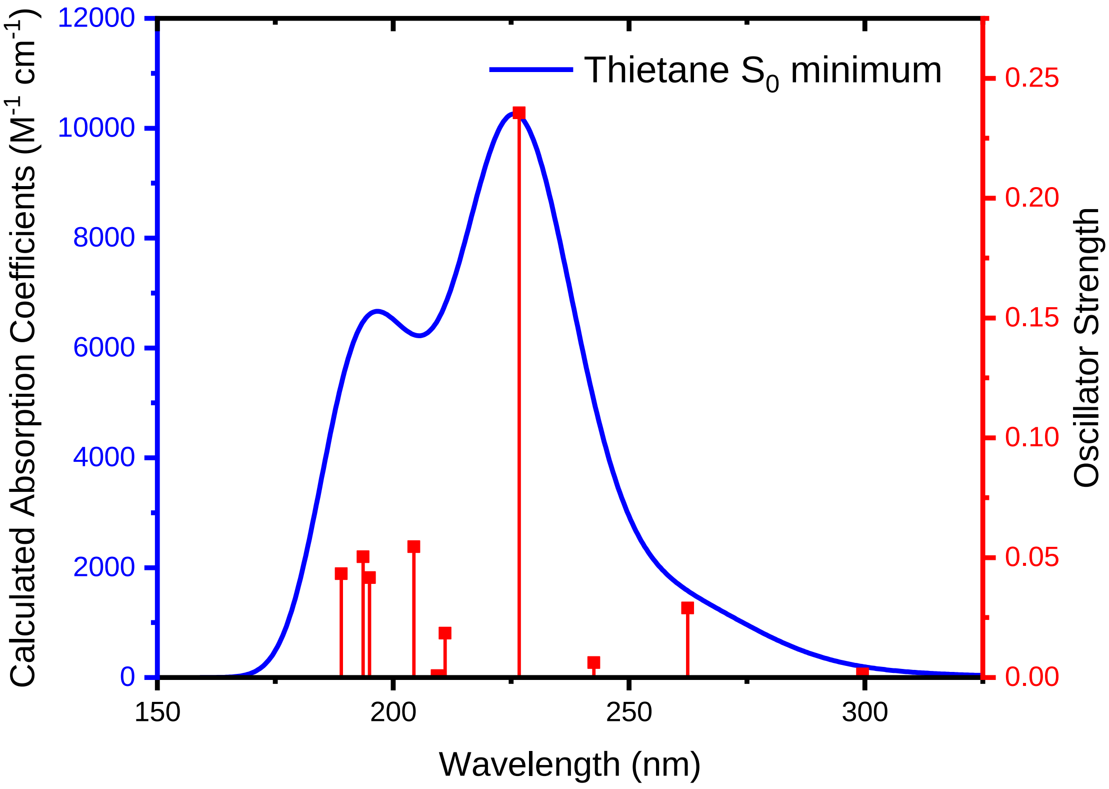

Supplementary Figure 5. Calculated absorptivity spectrum of the thietane minimum in water at the TD-M052X/IEFPCM/6-311++G(d,p) level of theory. In order to plot the results obtained, each transition was convoluted by a Gaussian with a FWHM of 0.33 eV. The optimized structure of the thietane minimum was taken from recent work published by Xie *et al.*,[8] which was calculated at the QM(CASSCF)/MM level of theory.

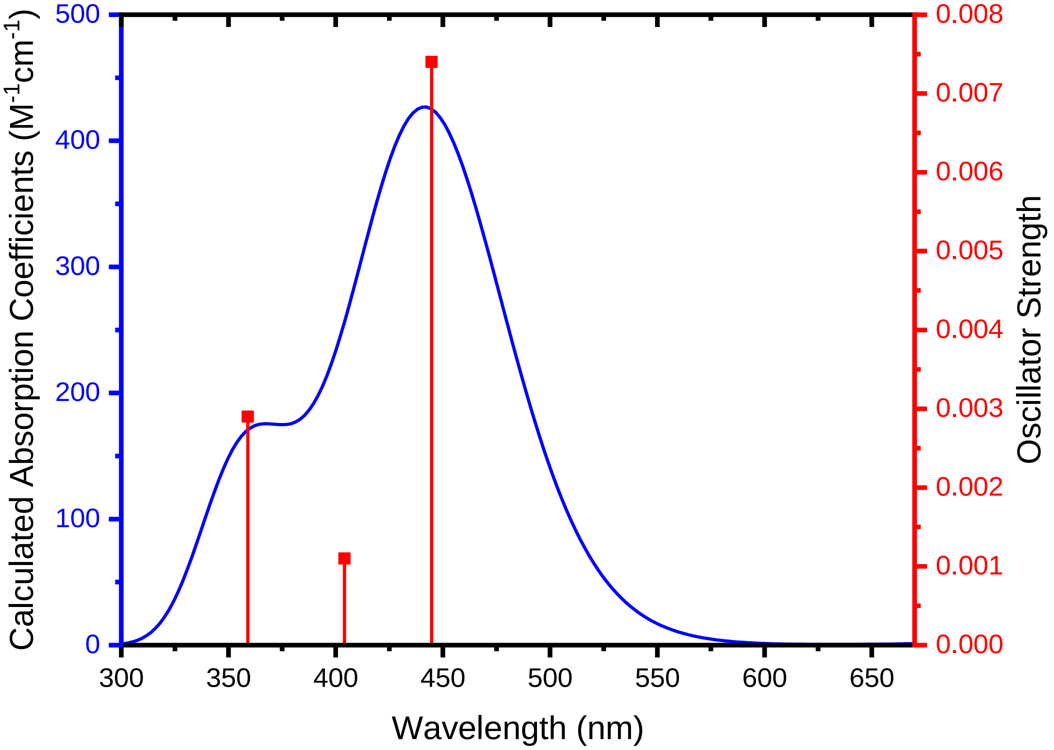

Supplementary Figure 6. Calculated absorptivity spectrum of the triplet-state minimum of the thietane (T_{1CC}) in water at the TD-UM052X/IEFPCM/6-311++G(d,p) level of theory. In order to plot the results obtained, each transition was convoluted by a Gaussian with a FWHM of 0.33 eV. The two lowest energy bands of T_{1CC} have maxima around 442 and 362 nm. The 442 nm band mainly stems from a ππ* transition from $T_0 \rightarrow T_2$, while the 362 nm band mainly stems from a ππ* transition from $T_0 \rightarrow T_4$. An intermediate transition $T_0 \rightarrow T_3$ is also observed with smaller oscillator strength. The VEE for the first four triplet states and the Kohn-Sham orbitals relevant to each transition of T_{1CC} are shown in Supplementary Table 1 and Supplementary Figure 10, respectively. The optimized structure of the T_{1CC} was taken from a recent work published by Xie *et al.*,[8] which was calculated at the QM(CASSCF)/MM level of theory. An unreal triplet transition with an energy of -0.19 eV and an oscillator strength of 0.00 was calculated at the TD-UM052X/IEFPCM/6-311++G(d,p) level of theory, which is not included in Supplementary Table 1 or in the absorption spectrum.

Supplementary Table 1. Vertical excitation energies for the triplet minimum of the thietane intermediate (T_{1CC}) calculated at the TD-UM052X/IEFPCM/6-31+G(d,p) level of theory in water. All the orbitals involved in the relevant transitions are beta-orbitals (HOMO = H, LUMO = L, and charge transfer = CT).

**State**	**Transitions**	**% Contribution**	**Character**	**eV**
**T_1**	H-9 → L+0	1.19	CT($n\pi$)*	1.37 (0.0003)
	H-6 → L+0	1.43	CT($n\pi$)*	
	**H-2 → L+0**	**5.09**	CT($\pi\pi$)*	
	H-1 → L+0	1.35	($n\pi$)*	
	**H-0 → L+0**	**90.94**	($n\pi$)*	
**T_2**	H-9 → L+0	3.69	CT($n\pi$)*	2.79 (0.0074)
	H-6 → L+0	2.32	CT(nπ)*	
	H-5 → L+0	2.56	CT($\pi\pi$)*	
	H-3 → L+0	5.16	CT(nπ)*	
	**H-2 → L+0**	**75.45**	CT($\pi\pi$)*	
	**H-0 → L+0**	**10.83**	($n\pi$)*	
**T_3**	**H-7 → L+1**	**8.33**	CT($n\pi$)*	3.07 (0.0011)
	H-5 → L+0	1.38	CT($n\pi$)*	
	**H-5 → L+1**	**10.57**	CT($\pi\pi$)*	
	**H-3 → L+0**	**9.27**	CT($n\pi$)*	
	**H-3 → L+1**	**66.60**	($n\pi$)*	
	H-2 → L+0	1.19	CT($n\pi$)*	
	H-0 → L+1	2.67	CT($\pi\pi$)*	
**T_4**	H-5 → L+0	2.62	CT($\pi\pi$)*	3.45 (0.0029)
	H-5 → L+1	2.37	($\pi\pi$)*	
	H-2 → L+1	4.41	($\pi\pi$)*	
	H-1 → L+0	1.34	($n\pi$)*	
	**H-1 → L+1**	**59.99**	CT($\pi\pi$)*	
	**H-0 → L+1**	**27.85**	CT($\pi\pi$)*	
	H-0 → L+2	1.41	($\pi\pi$)*	

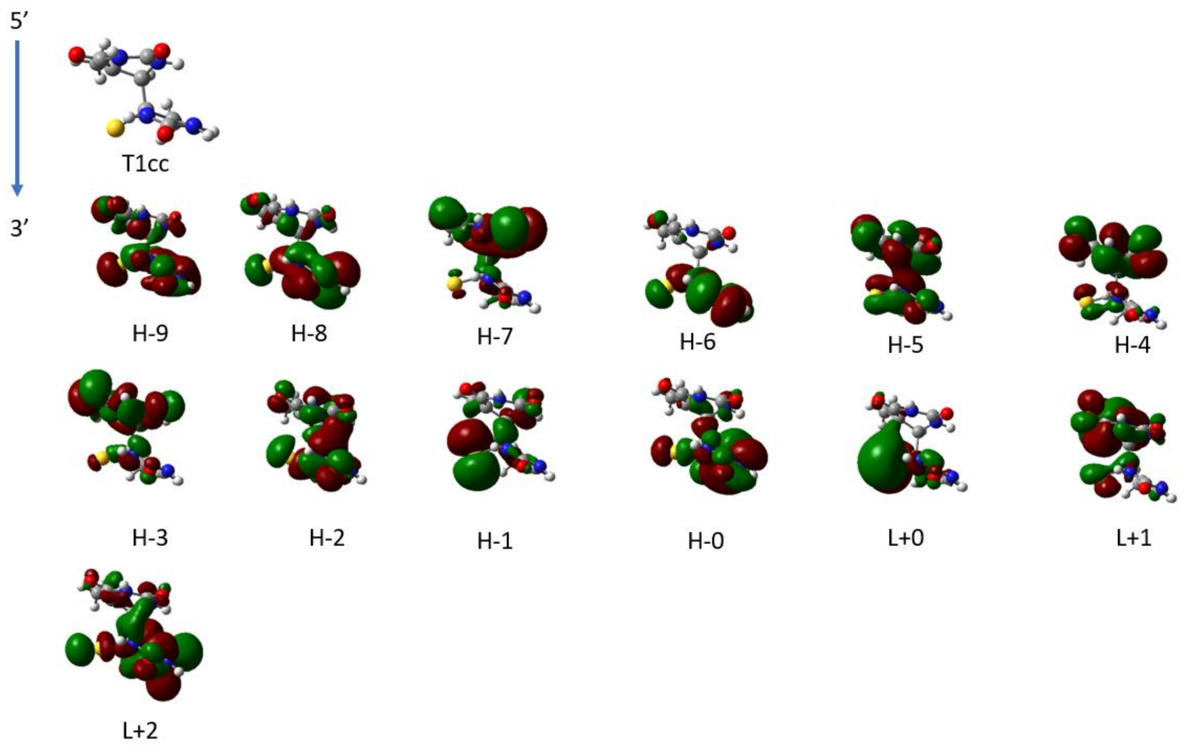

Supplementary Figure 7. Kohn-Sham orbitals that contribute to the relevant vertical transitions of T_{1CC} at the TD-UM052X/IEFPCM/6-311++G(d,p) level of theory in water. All the orbitals involved in the relevant transitions are beta-orbitals.

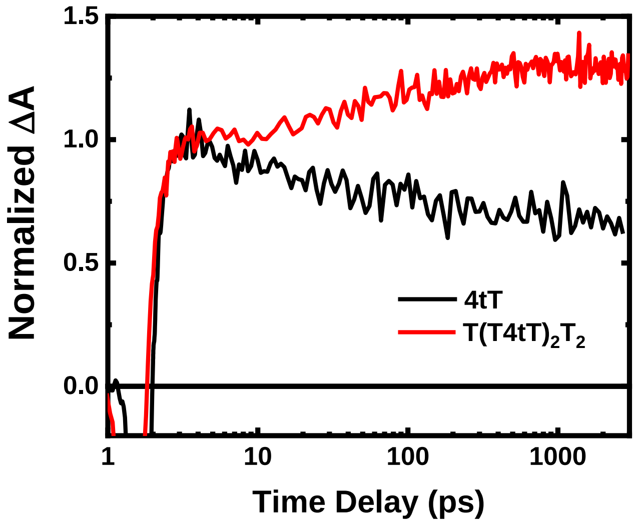

Supplementary Figure 8. Normalized decay traces of 4tT and $T(T4tT)_2T_2$ at 430 nm probe wavelength.

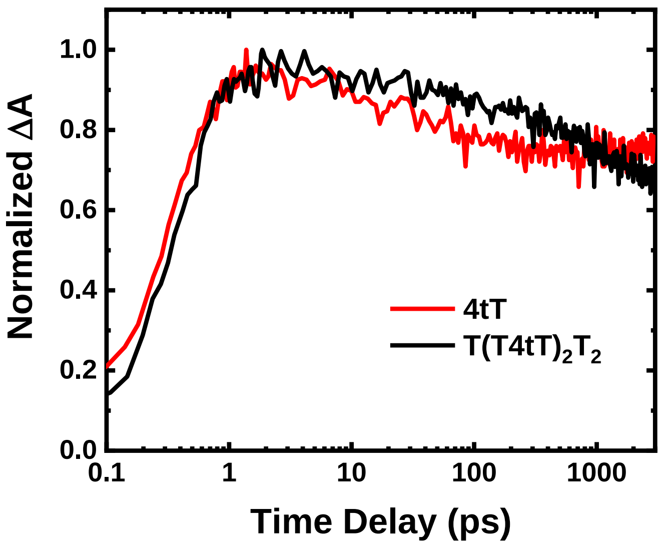

Supplementary Figure 9. Normalized decay traces of 4tT and $T(T4tT)_2T_2$ at 550 nm probe wavelength.

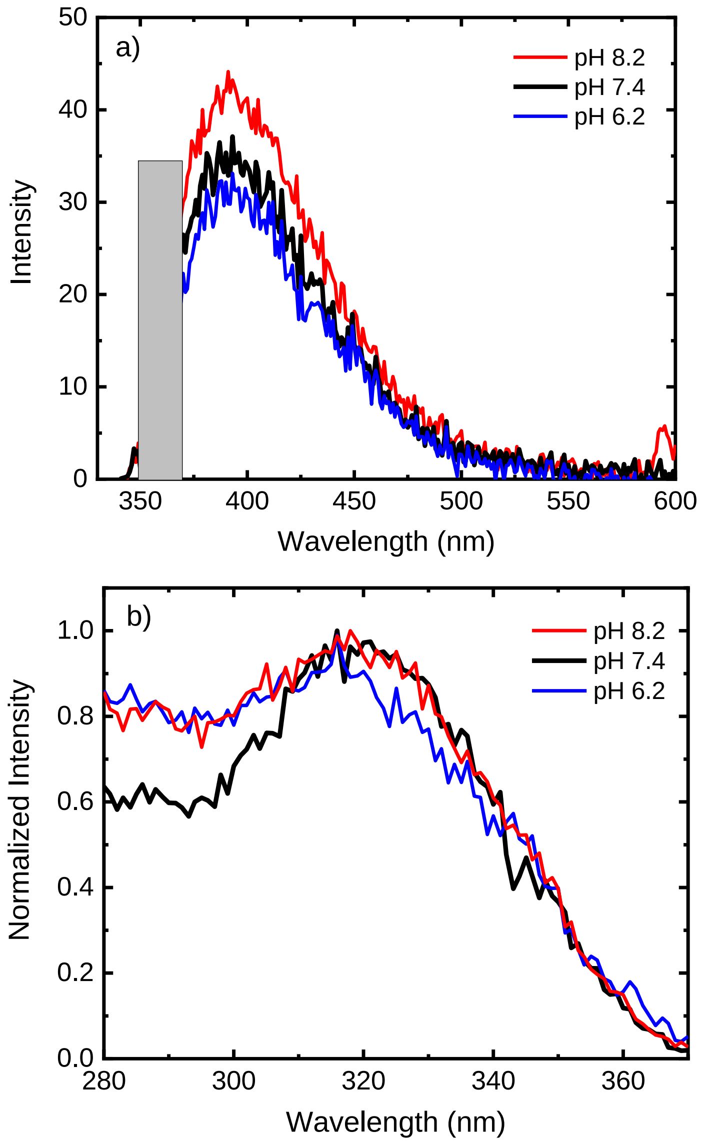

Supplementary Figure 10. (a) Emission spectra of the irradiated T(T4tT)$_2$T$_2$ oligonucleotide at different pHs (λ_{exc} = 320 nm). Raman signal from the solvent is masked with a gray rectangle. (b) Excitation spectra of the irradiated T(T4tT)$_2$T$_2$ oligonucleotide (λ_{em} = 390 nm). To generate these results, the oligonucleotide solution was irradiated at pH 7.4 for 35 min at 342 nm, followed by the collection of the emission spectra exciting at 320 nm in neutral (7.4), acidic (6.2), and basic (8.2) pH conditions. The pH of the PBS was adjusted with 0.1 M HCl and 0.1 M NaOH and the total phosphate concentration in the PBS is 16 mM in all three cases. We select a pH range from 6.2 to 8.2 in order to maintain the buffering capacity of the PBS and ensure that the pH stayed constant after adding small volume aliquots. To maintain the same concentration of the irradiated oligonucleotide at each pH conditions, three aliquots of 20 μL each were extracted from the irradiated sample at neutral pH. Each independent aliquot was added to 3 mL of PBS at the 3 different pHs for a total volume of 3.2 mL in a 1 cm optical cell, resulting in the acid, basic and neutral solutions used for the steady-state spectroscopic measurements.

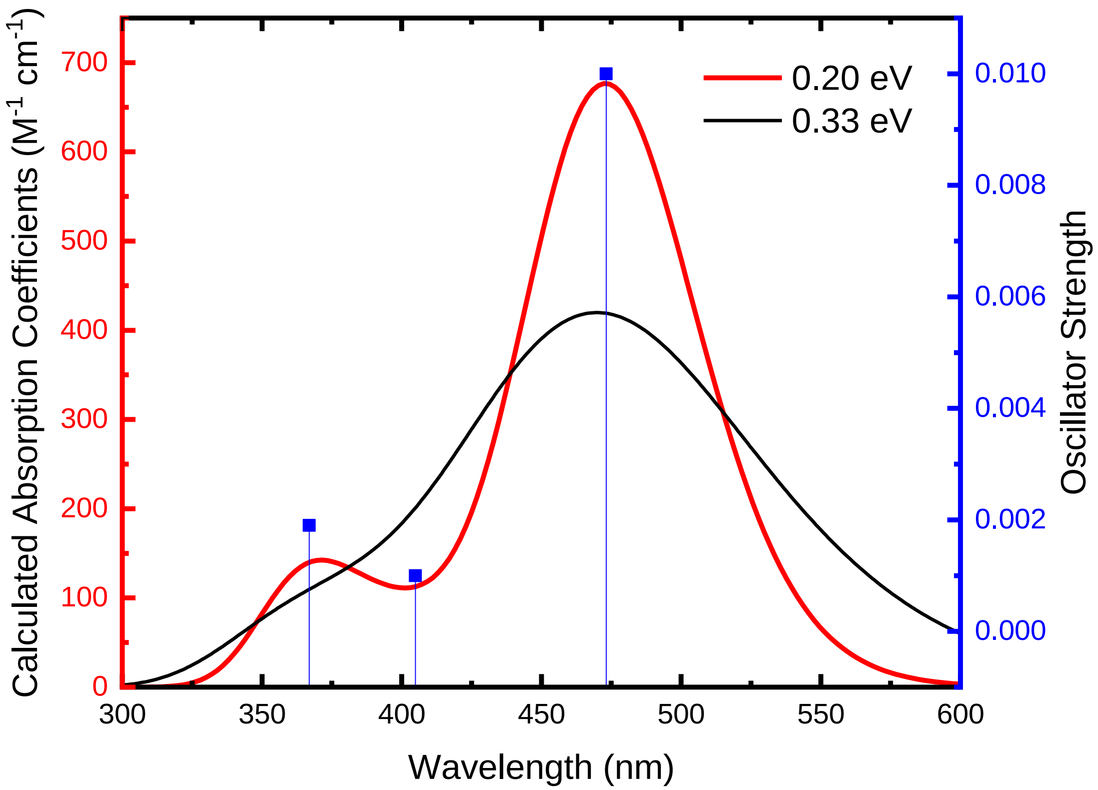

Supplementary Figure 11. Calculated absorption spectrum of T_{1CC} in water at the TD-UωB97xD/IEFPCM/6-311++G(d,p) level of theory. In order to plot the results obtained, each transition was convoluted by a Gaussian with a FWHM of 0.2 or 0.33 eV. The two lowest energy bands of T_{1CC} have maxima around 474 nm and 374 nm. The 474 nm band is mainly due to $T_0 \rightarrow T_2$, while the 374 nm band is mainly due to $T_0 \rightarrow T_4$. This is consistent with the results obtained with the M052X where the lowest energy band is due to $T_0 \rightarrow T_2$ and the higher energy band is due to $T_0 \rightarrow T_4$. The VEE for the relevant triplet states are shown in Table S2. The ground-state optimized structure of the T_{1CC} was taken from recent work published by Xie *et al.*,[8] which and was calculated at the QM(CASSCF)/MM level of theory.

Supplementary Table 2. Vertical excitation energies for the T_{1CC} thietane minimum calculated at the TD-UωB97xD /IEFPCM/6-31+G(d,p) level of theory in water

State	ωB97xD
**T_1**	1.30 (0.0003)
**T_2**	2.62 (0.0100)
**T_3**	3.06 (0.0010)
**T_4**	3.38 (0.0019)

Supplementary References

1. Warren, M. A., Murray, J. B. & Connolly, B. A. Synthesis and characterization of oligodeoxynucleotides containing thio analogues of (6-4) pyrimidine-pyrimidinone photo-dimers. *J. Mol. Biol.* **279**, 89-100 (1998).
2. Liu, J. & Taylor, J.-S. Remarkable photoreversal of a thio analog of the Dewar valence isomer of the (6-4) photoproduct of DNA to the parent nucleotides. *J. Am. Chem. Soc.* **118**, 3287-3288 (1996).
3. Ai, Y.-J., Liao, R.-Z., Chen, S.-F., Luo, Y. & Fang, W.-H. Theoretical studies on photoisomerizations of (6-4) and Dewar photolesions in DNA. *J. Phys. Chem. B* **114**, 14096-14102 (2010).
4. Yang, Z. B., Eriksson, L. A. & Zhang, R. B. A theoretical rationale for why azetidine has a faster rate of formation than oxetane in TC(6-4) photoproducts. *J. Phys. Chem. B* **115**, 9681-9686 (2011).
5. Vendrell-Criado, V., Rodrígues-Muñiz, G. M., Cuquerella, M. C., Lhiaubet-Vallet, V. & Miranda, M. A. Photosensitization of DNA by 5-methyl-2-pyrimidone deoxyribonucleoside: (6-4) photoproduct as a possible Trojan hourse. *Angew. Chem. Int. Ed.* **125**, 6604-6607 (2013).
6. Schreier, W. J., Gilch, P. & Zinth, W. Early Events of DNA Photodamage. *Annu. Rev. Phys. Chem.* **66**, 497-519 (2015).
7. Vendrell-Criado, V., Rodrígues-Muñiz, G. M., Lhiaubet-Vallet, V., Cuquerella, M. C. & Miranda, M. A. The (6-4) dimeric lesion as a DNA photosensitizer. *ChemPhysChem* **17**, 1979-1982 (2016).
8. Xie, B.-B. & Cui, C.-X. Theoretical studies on photo-induced cycloaddition and (6-4) reactions of the thymidine:4-thiothymidine dimer in a DNA duplex. *Phys. Chem. Chem. Phys.* **21**, 2006-2016 (2019).
